# A Screen-Printed Electrode Modified With Graphene/Co_3_O_4_ Nanocomposite for Electrochemical Detection of Tramadol

**DOI:** 10.3389/fchem.2020.562308

**Published:** 2020-11-30

**Authors:** Mohammad Reza Aflatoonian, Somayeh Tajik, Behnaz Aflatoonian, Hadi Beitollahi, Kaiqiang Zhang, Quyet Van Le, Joo Hwan Cha, Ho Won Jang, Mohammadreza Shokouhimehr, Wanxi Peng

**Affiliations:** ^1^Neuroscience Research Center, Kerman University of Medical Sciences, Kerman, Iran; ^2^Leishmaniasis Research Center, Kerman University of Medical Sciences, Kerman, Iran; ^3^Research Center for Tropical and Infectious Diseases, Kerman University of Medical Sciences, Kerman, Iran; ^4^Environment Department, Institute of Science and High Technology and Environmental Sciences, Graduate University of Advanced Technology, Kerman, Iran; ^5^Department of Materials Science and Engineering, Research Institute of Advanced Materials, Seoul National University, Seoul, South Korea; ^6^Institute of Research and Development, Duy Tan University, Da Nang, Vietnam; ^7^Innovative Enterprise Cooperation Center, Korea Institute of Science and Technology, Seoul, South Korea; ^8^College of Forestry, Henan Agricultural University, Zhengzhou, China; ^9^School of Automotive Engineering, Huanghe Jiaotong University, Jiaozuo, China

**Keywords:** electrochemical sensor, tramadol, graphene/Co_3_O_4_ nanocomposite, screen printed electrode, nanomaterials

## Abstract

In this paper, graphene (Gr)/Co_3_O_4_ nanocomposite was synthesized and utilized for the development of a novel electrochemical sensor to detect tramadol. Tramadol determination was examined by linear sweep voltammetry, differential pulse voltammetry, cyclic voltammetry, and chronoamperometry on Gr/Co_3_O_4_ nanocomposite-modified screen-printed electrode (Gr/Co_3_O_4_/SPE) in phosphate-buffered saline (PBS). Under the optimized condition, the detection limit of tramadol is 0.03 μM (S/N = 3) in the linear ranges of 0.1–500.0 μM. Furthermore, Gr/Co_3_O_4_/SPE was satisfactorily utilized to detect tramadol in tramadol tablet and urine specimens.

## Introduction

Tramadol is a synthetic analog of codeine that acts as a central analgesic. In terms of structure, there is a relationship between tramadol and codeine and morphine. Moreover, it acts as one of the opioid agonists in the body (Abu-Shawish et al., 2010). In addition, it is possible to use tramadol alone or combined with additional non-steroidal anti-inflammatory drugs (NSAIDs) when dealing with patients who have serious chronic pain, depression, spinal cord injuries, low back pain, and post-operative pain (Tetsunaga et al., 2015). Furthermore, it is increasingly being abused by opioid addicts. However, usual dosing requirements for oral consumption of the drug ranges between 50 and 100 mg per 4–6 h, with the maximum dose being 400 mg a day (Thévenin et al., 2008). Overdosing on tramadol can cause vomiting, problems in the central nervous system (CNS) and respiratory system, depression, dizziness, nausea, coma, tachycardia, and seizure (Scott and Perry, 2000). Tramadol is rapidly and nearly completely absorbed in oral usage, but excretion of roughly 10–30% of the parent drug happens un-metabolized in urine (Mohammadpour et al., 2019). Thus, for reasons related to treatment and the drug, it is of special significance to propose a sensitive technique to measure specimens containing of tramadol. At present, many methods have been employed for measuring tramadol quantitatively, such as high-performance liquid chromatography (Kmetec and Roškar, 2003; Saccomanni et al., 2010), gas chromatography (Ghasemi, 2012), gas chromatography/mass spectrometry (El-Sayed et al., 2013), electrochemiluminescence (Ding et al., 2006), spectrophotometry (Glavanović et al., 2016), capillary electrophoresis (Cunha et al., 2017), and electrochemistry (Ghorbani-Bidkorbeh et al., 2010; Babaei et al., 2011; Chitravathi and Munichandraiah, 2016; Madrakian et al., 2017; Hassannezhad et al., 2019; Hassanvand and Jalali, 2019; Rokhsefid and Shishehbore, 2019).

Nevertheless, some of these methods are limited due to complex sample procurement stages and the utilization of organic solvents. Electrochemical techniques have been proposed instead when analyzing drug and biological specimens because of the simplification, speed, inexpensive instruments, higher sensitivity, and precise analytical devices (Mahmoudi-Moghaddam et al., 2014; Prasad and Sreedhar, 2018; Elobeid and Elbashir, 2019; Tan et al., 2020; Xia et al., 2020). Considering the above features to detect diverse significant samples, researchers utilized many procedures for improving the electrode modification in order to enhance selectivity and sensitivity (Beitollahi and Sheikhshoaie, 2012; Soltani et al., 2014; Zhang et al., 2015; Deshmukh et al., 2018; Ghodsi et al., 2018; Mohammadi et al., 2018; Rabiee et al., 2018).

Nanomaterials are defined as substances possessing at least one dimension with ~100 nanometers. They are very interesting materials because of their unique electrical, magnetic, and optical, properties. These emergent features provide the potential for great impacts in medicine, electronics, and other fields (Zhu et al., 2015; Abdussalam-Mohammed, 2019; Elumalai et al., 2019). Now, interest has grown in using nanomaterials for modifying the electrode surface. However, owing to nanomaterials' very good catalytic features and conductivity, we can use them for enhancing the electron transfer between the electrode surface and target analyte. Moreover, they can be used as the catalyst for increasing the electrochemical reaction (Beitollahi et al., 2008, 2018; Li et al., 2010, 2016; Yang et al., 2014; Moghaddam et al., 2015; Zheng et al., 2018).

In this regard, Gr is an ideal nanomaterial for electrochemical processes owing to its larger surface area, reasonable electrical conductivity, and inexpensiveness (Shao et al., 2010). In the fabrication of the electrochemical sensors and biosensors, the Gr-based electrodes have been considered interesting films for the sensing platforms as they cause synergy of the electrocatalytic activities and augment the sensor sensitivity (Zhang et al., 2011). In fact, the Gr sheets are very good host materials to make nanocomposites for high performance electrochemical utilizations (Kim et al., 2009; Zhou et al., 2010; Vinodhkumar et al., 2018). Gr nanocomposites display acceptable benefits as one of the sensing platforms in electrochemical sensors (Luo et al., 2012; Thanh et al., 2016; Shahid et al., 2019). Combining the advantages of the unique features of Gr together with those ofCo_3_O_4_ (the increased absorption capability, larger active site, and wide availability), the Gr/Co_3_O_4_ nanocomposite provides stable and sensitive platforms for electroanalysis (Yavuz et al., 2013a,b; Feng et al., 2014). Therefore, we have used Gr/Co_3_O_4_ as one of the nanocomposite materials because it has the most reasonable features.

According to studies, the screen-printing technology used for microelectronics has a significant utilization for fabricating electrodes for disposable electrochemical sensors and biosensors (Tangkuaram et al., 2007; Gevaerd et al., 2020). In fact, an SPE enjoys simplified operation, versatility, inexpensiveness, portability, reliability, and lower sizes, and has the capability for mass production. Hence, it has widespread application in the field of electroanalytical chemistry (Nicholas et al., 2018; Khalilzadeh et al., 2020). Moreover, an SPE would avoid the cleaning procedure in contrast to the traditional electrodes, like a glassy carbon electrode (Renedo et al., 2007). SPE resolves the shortcomings of the traditional electrode systems, which require repeated recalibration and are not stable and suitable for on-site analyses, because their completion lasts several hours. Additionally, they require the use of professionals, as they need multiple isolation and washing phases. Therefore, the mentioned disadvantages of the traditional electrode systems resulted in their lower capability in comparison to the SPEs.

We aimed at providing a technique with higher simplification, sensitivity, and speed to electrochemically detect tramadol. Therefore, a Gr/Co_3_O_4_/SPE was fabricated by dropping Gr/Co_3_O_4_ over SPE to make a voltametric sensor as well as evaluating tramadol voltammetric behaviors. According to our analysis, Gr/Co_3_O_4_/SPE exhibited more robust electrochemical oxidation for tramadol with a more negative potential. The present method was tested and verified using tramadol tablet and urine specimens, showing reasonable precision and recovery. To the best of our knowledge, the detection of tramadol by using Gr/Co_3_O_4_/SPE has not been reported yet.

## Experimental

### Apparatus and Chemicals

Autolab potentiostat/galvanostat (PGSTAT 302N, Eco Chemie, the Netherlands) was used to measure electrochemicals. The use of General Purpose Electrochemical System (GPES) software aimed at controlling the experimental conditions. Moreover, SPE (DropSens; DRP-110: Spain) possessed three typical graphite counters, unmodified graphite working, and silver pseudo-reference electrodes. Metrohm 710 pH meter was utilized for pH measurements.

Tramadol and other remaining reagents represented an analytical grade. Their preparation was carried out through Merck (Darmstadt, Germany). Orthophosphoric acid and the associated salts with a pH ranging higher than 2.0–9.0 have been utilized to prepare the buffer solutions.

### Synthesizing the Gr/Co_3_O_4_ Nanocomposite

The synthesis of the Gr/Co_3_O_4_ composite was done via the chemical deposition of Co_3_O_4_ particles over the graphene oxide (GO), which was accompanied by reducing GO to Gr in NaBH_4_ solution. It is notable that Li et al. fixed synthesis and composition of the Gr/Co_3_O_4_ composite (Li et al., 2011). Therefore, we poured the graphite oxide (0.1 g) into 200 mL of ultra-high purity water and placed it in an ultrasonic bath for 2 h in order to establish a GO suspension. Then, an aqueous solution (10 mL) of CoCl_2_ (1.4 g CoCl_2_.6H_2_O) was added in this suspension. The mixture was shaken for two hours for completion of the ion exchange. The next stage concerned the drop wise addition of an aqueous solution (10 mL) of NaOH (1 equivalent) to the obtained mix by shaking for another hour. Afterwards, we achieved the solid using centrifugation via the ultrahigh purity water. Next, the product was distributed into ultra-high pure water (25 mL). Hydrogen peroxide (30%, 1.5 mL) was added to the obtained mix. This mix has been sealed into a 50 mL Teflon-lined stainless-steel autoclave. Then, heating was performed to 100°C and it was kept at the mentioned temperature for four hours. Notably, we collected the solid product through centrifugation that had been accompanied by a suspension in the ultra-high purity water (25 mL). In addition, we added NaBH_4_ (0.25 g) into the obtained suspension, and transported it to the autoclave heated to 120°C. Finally, we kept it at the above temperature for four hours. Finally, we collected the obtained solid and used ultra-high purity water to wash it. The solid was dried to achieve the Gr/Co_3_O_4_ composite. The composite was gained as black powder.

### Preparation of Electrode

The bare SPE was coated by a Gr/Co_3_O_4_ nanocomposite. To prepare the Gr/Co_3_O_4_ nanocomposite stock solution in 1 mL of aqueous solution, the Gr/Co_3_O_4_ nanocomposite (1 mg) was distributed by 30-min ultra-sonication, whereas the Gr/Co_3_O_4_ nanocomposite suspension aliquots (5 μl) were cast on working electrodes. Then it was kept until the solvent was evaporated in room temperature.

### Preparation of Real Sample

Five 50 mg tramadol pills (Amin Co., Iran) were used to prepare a solution by dissolving the pills (250 mg) in water (25 ml) in exposure to ultra-sonication. Then, different dilutions were poured in 25 ml volumetric flasks and reached final volume with PBS at pH of 7.0. The standard addition method was used to measure the tramadol concentrations.

10 ml of directly refrigerated urine samples were centrifuged at 2,000 rpm for 15 min, followed by filtering the supernatant using a 0.45 μm filter. Then, different volumes of solution were distributed into a 25 ml volumetric flask and diluted with PBS (pH = 7.0) until the mark, which were anesthetized by various doses of tramadol. The standard addition method was used to determine the tramadol concentrations.

## Result and Discussion

### Characterization of Nanostructures

**Figure 1:**
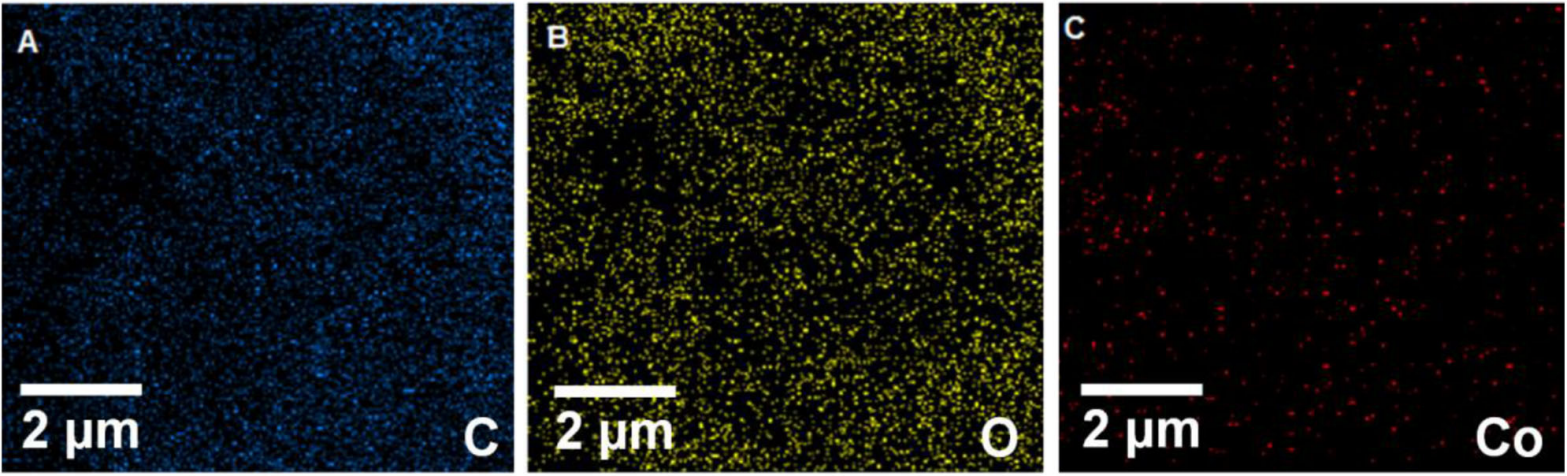
EDX elemental mapping of **(A)** carbon, **(B)** oxygen, and **(C)** cobalt in G/Co_3_O_4_ nanocomposite.

**Figure 2:**
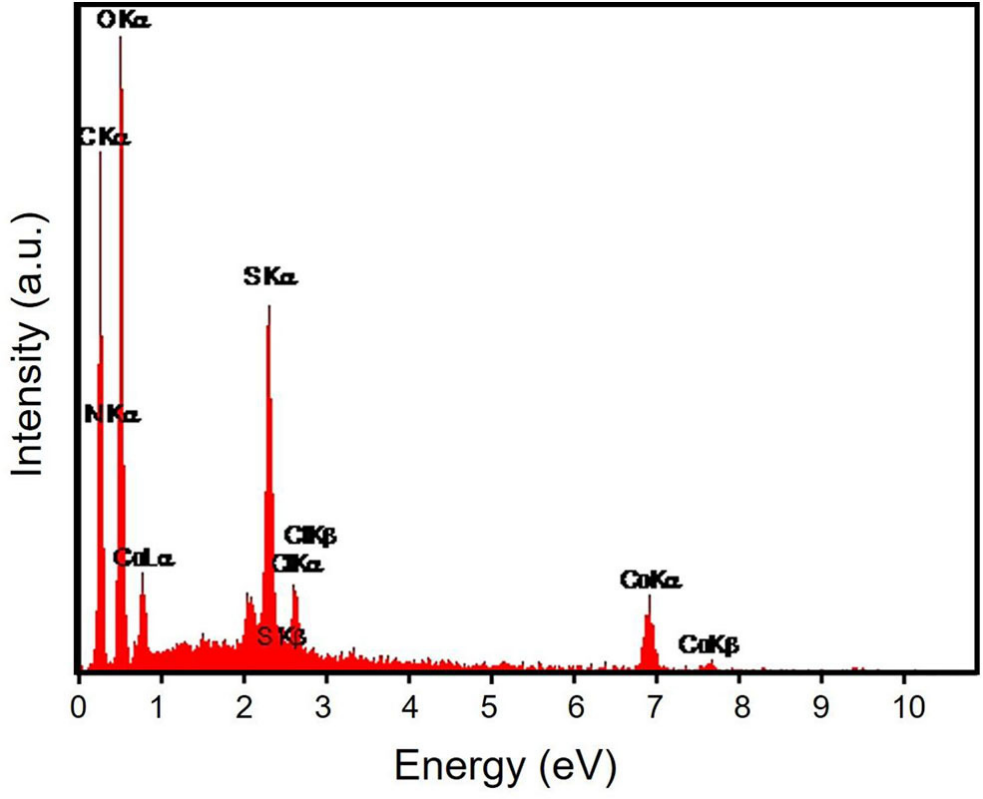
EDS spectrum of Gr/Co_3_O_4_ nanocomposite.

**Figure 3:**
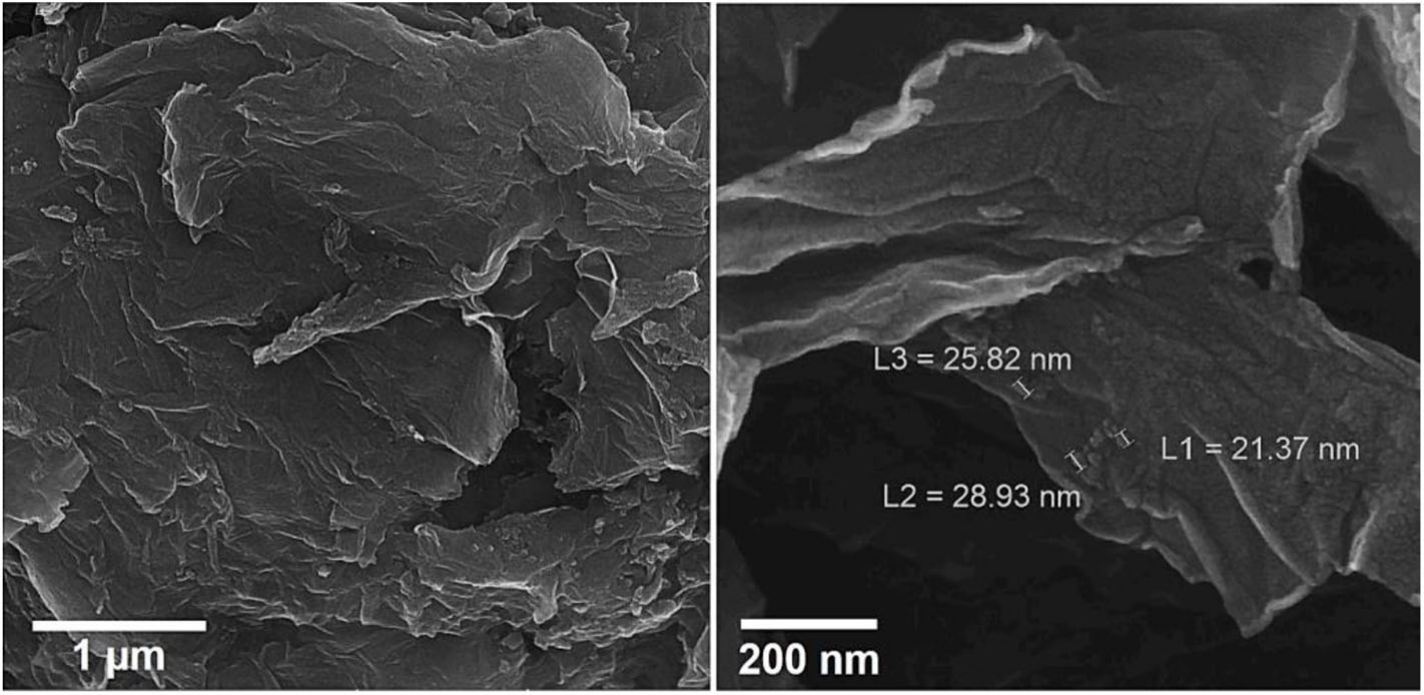
SEM images of Gr/Co_3_O_4_ nanocomposite.

The energy-dispersive X-ray spectroscopy (EDX) elemental mapping has been analyzed for the Gr/Co_3_O_4_ nanocomposite for the control of distributing the elements found in the nanocomposite (Figure 1). In order to have a clear result, Figures 1A–C shows the independent elemental distribution of C, O, and Co. Figure 1A represents the existence of GO as the carbon materials whereas Figure 1B (O) and Figure 1C (Co) show a big area coverage because of the densely packaged Co_3_O_4_ nanoparticles. The presence of all elements (oxygen, carbon, and cobalt) in the Gr/Co_3_O_4_ nanocomposite was confirmed by EDS analysis (Figure 2). We applied the scanning electron microscope (SEM) for characterizing the morphology of the as-synthesized Gr/Co_3_O_4_ nanocomposite. A SEM image of the Gr/Co_3_O_4_ nanocomposite is given in Figure 3.

### Electrochemical Behavior of Tramadol on the Gr/Co_3_O_4_/SPE

**Figure 4:**
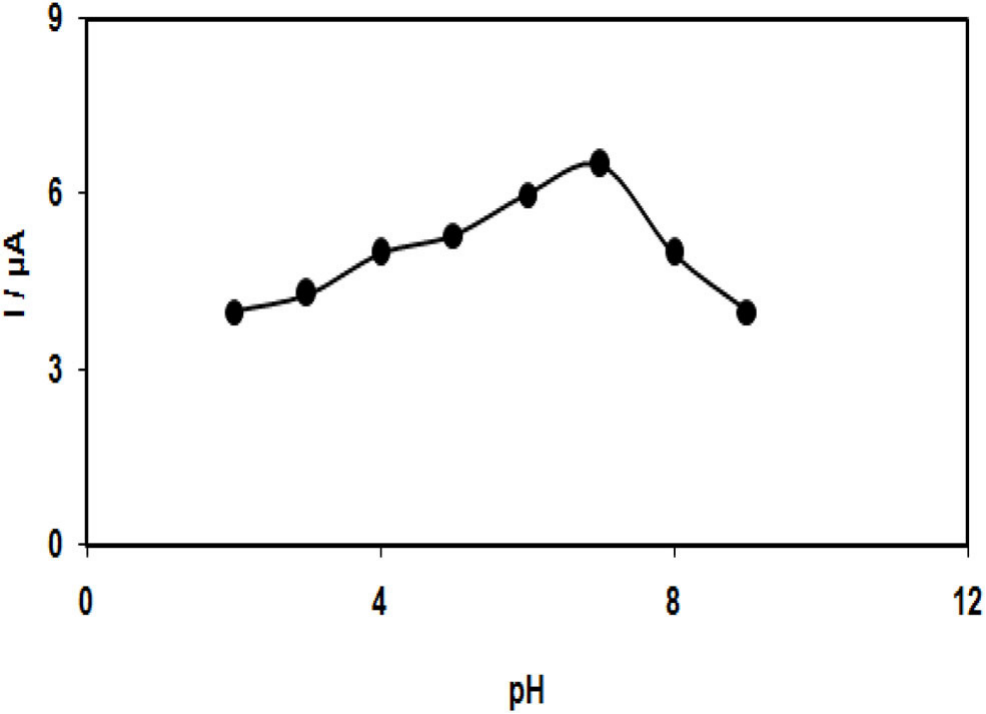
Plot of Ip *vs*. pHs of buffer solution (2.0, 3.0, 4.0, 5.0, 6.0, 7.0, 8.0, and 9.0) in the presence of 100.0 μM tramadol.

To study the electrochemical behaviors of tramadol, which was supposed to show dependence on pH, obtaining an optimum pH-value to reach acceptable outcomes is important. Thus, the modified electrode was employed in running the experiments with different pH values varying between 2.0 and 9.0. Eventually, the most desirable results were considered for electro-oxidation of tramadol at a pH of 7.0 (Figure 4).

**Figure 5:**
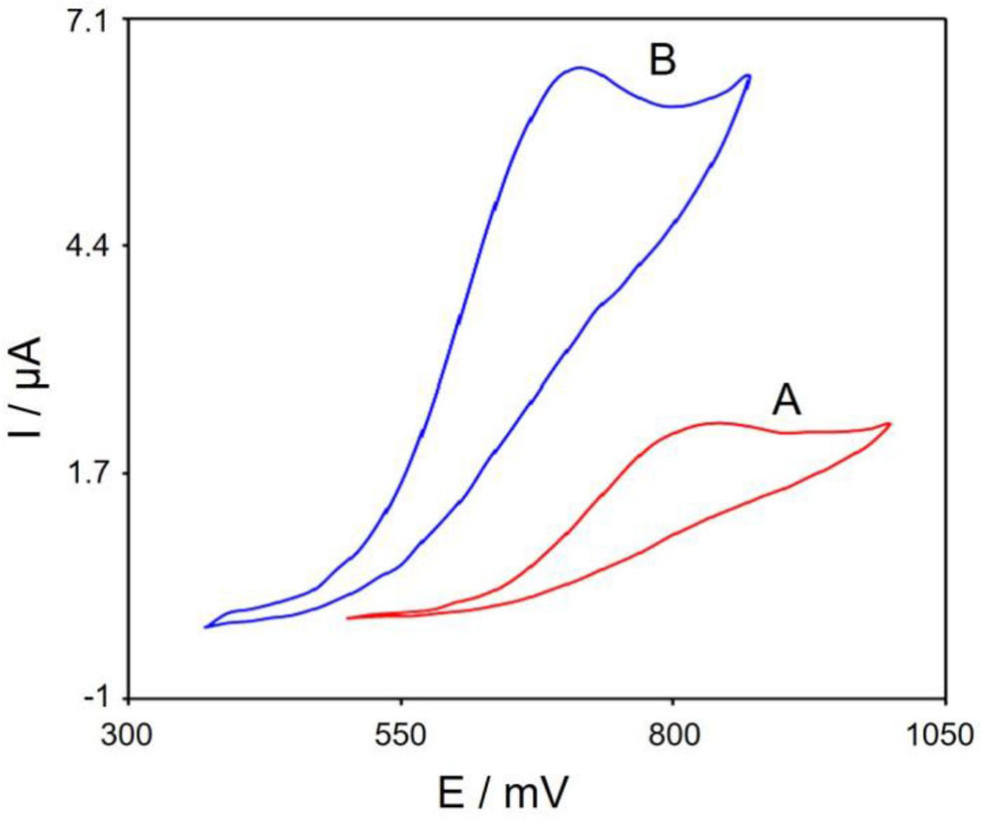
The CVs of **(A)** bare SPE, **(B)** Gr/Co_3_O_4_/SPE in 0.1 M PBS at pH of 7.0 when 100.0 μM tramadol is present at 50 mVs^−1^ scan rate.

Figure 5 represents cyclic voltammograms in the presence of 100.0 μM tramadol with the bare SPE (Curve a) and Gr/Co_3_O_4_/SPE (Curve b). Based on the CV outputs, the greatest oxidation of tramadol on the Gr/Co_3_O_4_/SPE occurred at 700 mV, which was ~140 mV more negative than the bare SPE.

### The Scan Rate Effects on the Results

**Figure 6:**
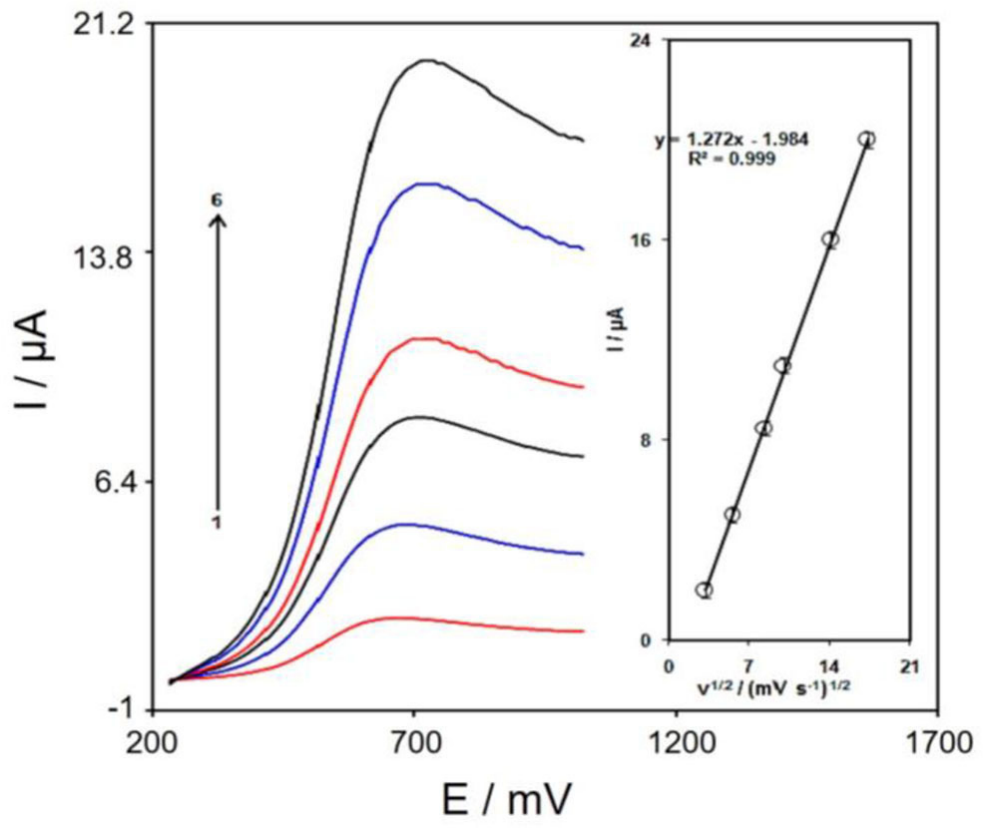
LSVs of Gr/Co_3_O_4_/SPE in 0.1 M PBS at pH of 7.0 including 150.0 μM tramadol at distinct scan rates; 1-6 respective to 10, 30, 70, 100, 200, and 300 mV s^−1^. Inset; variations in the anodic peak currents vs. ν^1/2^.

The association between peak current and scan rate would supply helpful information considering the electrochemical mechanisms. Therefore, the scan rate effects on the peak current of tramadol were examined using LSV, at a range of 10–300 mVs^−1^ in PBS (0.1 M, pH 7), according to Figure 6. The electrode response of tramadol was a diffusion-controlled procedure, as the oxidation peak current corresponded to the square root of the scan rate (Figure 6 inset).

### Chronoamperometric Analysis

**Figure 7:**
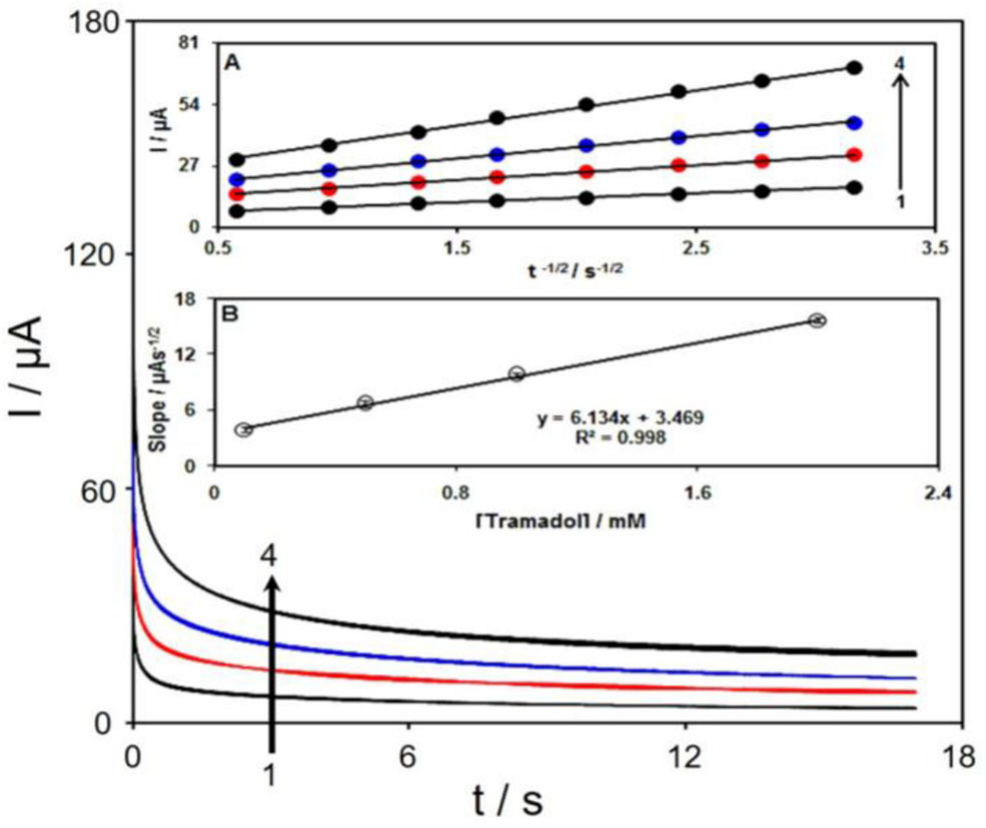
The chronoamperograms obtained at Gr/Co_3_O_4_/SPE in 0.1 M PBS at pH of 7.0 for tramadol at various concentrations. Accordingly, 1–4 relate to 0.1, 0.5, 1.0, and 2.0 mM of tramadol. Inset **(A)** the I plot vs. t^−1/2^ observed using chronoamperograms 1–5. **(B)** The straight-line slope plot vs. tramadol concentration.

Chronoamperometric study was used to calculate the diffusion coefficient (D) of tramadol at the surface Gr/Co_3_O_4_/SPE at an optimum condition. Figure 7 displays the chronoamperometric outputs of the tramadol sample at different concentrations (PBS at pH of 7.0). In addition, Cottrell equation was recommended to perform electroactive moiety chronoamperometric analyses according to the mass transfer restricted conditions (Bard and Faulkner, 2001):

$$\begin{array}{l} I=nFAD^{1/2}C_{\text{b}}\pi^{-1/2}t^{-1/2} \end{array}$$

Figure 7A indicates experimental findings regarding I vs. t^−1/2^, which shows the most acceptable fit for tramadol distinct concentrations. Then, the ultimate slopes relative to the straight lines in Figure 7A could be depicted vs. tramadol concentrations (Figure 7B). Thus, D mean-value equaled to 1.3 × 10^−5^ cm^2^/s with regard to Cottrell equation and resultant slopes.

### Calibration Curve

**Figure 8:**
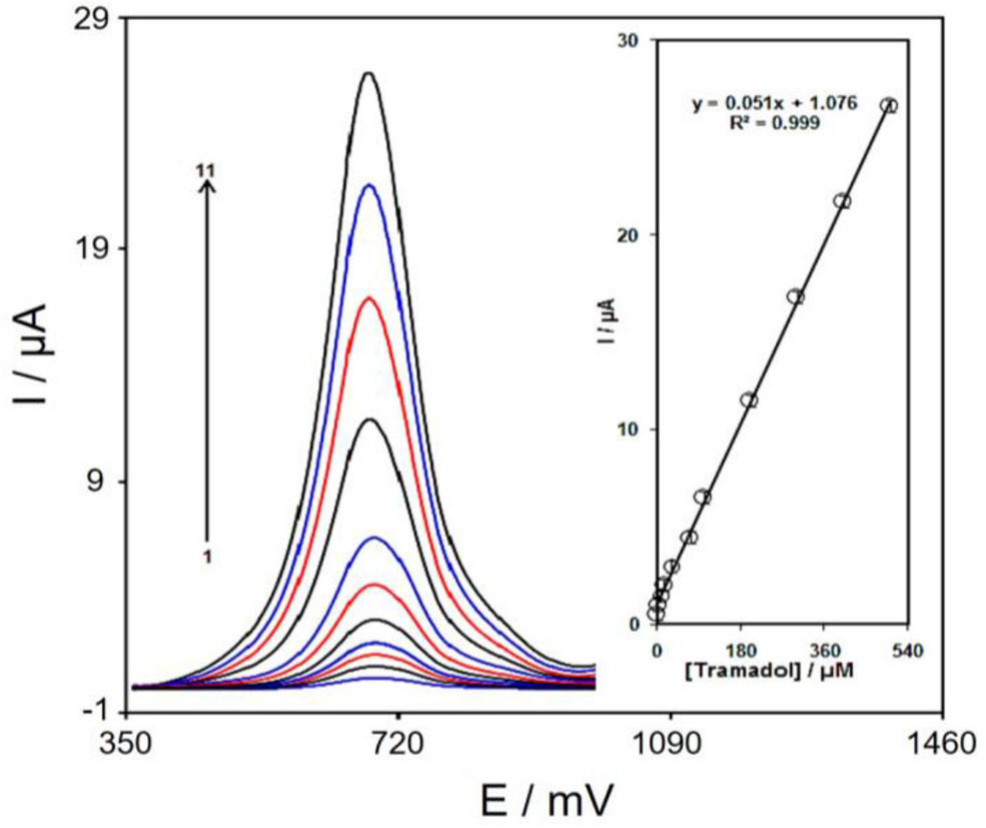
DPVs of Gr/Co_3_O_4_/SPE in 0.1 M PBS (pH 7.0) including tramadol at varying concentrations. 1–11 indicate 0.1, 2.0, 7.5, 15.0, 30.0, 70.0, 100.0, 200.0, 300.0, 400.0, and 500.0 μM of tramadol, respectively. The inset indicates the peak current plot as a function of the tramadol concentrations ranging from 0.1–500.0 μM.

The DPV method explored the association of the peak current with tramadol at different concentrations. As shown in Figure 8, the DPVs of Gr/Co_3_O_4_/SPE in the presence of different concentrations of tramadol was recorded in the concentration ranging from 00.5 to 500.0 μM. The detection limit 30.0 nM was established for analysis of tramadol at the surface of Gr/Co_3_O_4_/SPE. The response time was ~13 s.

The comparison study of the results for the determination of tramadol with different modified electrodes in the literature are listed in Table 1. The novel sensor presented in this study exhibits outstanding analytical performance for the determination of tramadol. The comparative data showed excellent efficiency of the proposed sensor over some earlier reported sensors. Thus, the fabricated electrode was appropriate for the determination of tramadol with the advantages of facile fabrication, ease of use, and low cost.

**Table 1:** Comparing performances of the purposed electrochemical sensor with others for determination of tramadol.

**Electrochemical sensor**	**Method**	**Linear range (μM)**	**Limit of detection (μM)**	**References**
CNPs/GCE^a^	DPV	10–1000	5	Ghorbani-Bidkorbeh et al., 2010
MWCNTs/GCE^b^	DPV	2–300	0.36	Babaei et al., 2011
Au NPs/cysteic acid/GCE^c^	SWV^d^	0.5–63.5	0.17	Hassanvand and Jalali, 2019
g-C_3_N_4_/Fe_3_O_4_/CPE^e^	DPV	0.2–14.0 and 14.0–120.0	0.1	Hassannezhad et al., 2019
Au NPs/GrN/CPE^f^	DPV	1.0–100.0	0.82	Rokhsefid and Shishehbore, 2019
Magneto LDH/Fe_3_O_4_ NPs/GCE^g^	DPV	1–200	0.3	Madrakian et al., 2017
PNB/GCE^h^	DPV	1–310	0.5	Chitravathi and Munichandraiah, 2016
Gr/Co_3_O_4_/SPE	DPV	0.1–500.0	0.03	This work

a Carbon nanoparticles modified glassy carbon electrode.

b Multiwalled carbon nanotube modified glassy carbon electrode.

c Au nanoparticles/cysteic acid modified glassy carbon electrode.

d Square wave voltammetry.

e Graphitic carbon nitride/Fe_3_O_4_ nanocomposite modified carbon paste electrode.

f Au nanoparticles/graphene nanosheet modified carbon paste electrode.

g Magneto layer double hydroxide/Fe_3_O_4_ nanoparticles modified glassy carbon electrode.

h *Poly(Nile blue) modified glassy carbon electrode*.

### Stability and Repeatability of Gr/Co_3_O_4_/SPE

The stability of Gr/Co_3_O_4_/SPE was evaluated using records of the oxidation signal of 40.0 μM tramadol over 2 weeks. A 2.5% deviation was identified with compression of the first oxidation signal of tramadol after 2 weeks, indicating good stability of Gr/Co_3_O_4_/SPE as a voltammetric sensor.

Examination of the modified SPE anti-fouling features regarding tramadol oxidation and the corresponding products carried out through CV for the modified SPE prior and subsequent to application when tramadol was present. CVs were recorded when tramadol was present following cycling the potential 15 times at a 50 mV s^−1^. The currents were reduced by more than 2.3 %, while the peak potential faced no alterations.

### Analyzing the Real Samples

Finally, Gr/Co_3_O_4_/SPE performance as a new electrochemical sensor used to analyze tramadol in tramadol tablet and urine samples was evaluated. Table 2 indicates the collected data and, according to the recovery data, Gr/Co_3_O_4_/SPE could be regarded as a sensitive sensor to analyze tramadol in actual samples.

**Table 2:** The application of Gr/Co_3_O_4_/SPE for determination of tramadol in tramadol tablet and urine samples (*n* = 5).

**Sample**	**Spiked**	**Found**	**Recovery (%)**	**R.S.D. (%)**
**Tramadol tablet**	0	5	-	3.5
	2.5	7.3	97.3	2.7
	7.5	12.8	102.4	1.8
	12.5	17.9	102.3	2.3
	17.5	22.3	99.1	2.2
**Urine**	0	-	-	-
	5	5.1	102	2.7
	10	9.8	98	2.1
	15	15.5	103.3	3.4
	20	19.8	99	1.9

*All concentrations are in μM*.

## Conclusions

We used a simplified procedure to synthesize Gr/Co_3_O_4_ and characterized it by EDX and SEM. Then, it was utilized for the electrochemical detection of tramadol. The modified electrode exhibited good electrocatalytic activity and sensitivity. Linear response of its peak current on tramadol concentrations ranged between 0.1 and 500.0 μM, and LOD was 0.03 μM. Finally, the modified electrode was substantially used for tramadol analysis in the real specimens. The proposed method offers a sensitive approach to detect tramadol in drug and biological formulations.

## Data Availability Statement

The raw data supporting the conclusions of this article will be made available by the authors, without undue reservation.

## Author Contributions

All authors have contributed to the scientific discussion, investigation, analysis, and manuscript writing and editing.

## Conflict of Interest

The authors declare that the research was conducted in the absence of any commercial or financial relationships that could be construed as a potential conflict of interest.
